# The current crisis of academia-led research: a threat to the common good? Preliminary data from Europe and the United States

**DOI:** 10.1186/s13104-020-05128-9

**Published:** 2020-07-08

**Authors:** Mauro Giovanni Carta, Maria Francesca Moro, Iskren Kirilov, Ferdinando Romano, Silvano Tagliagambe

**Affiliations:** 1 Dipartimento di Medicina Interna e Salute Pubblica, University of Cagliari, Cagliari, Italy; 2 PhD Programme Innovation Sciences and Technologies, University of Cagliari, Cagliari, Italy; 3 Department of Public Health and Infectious Diseases, University of Rome La Sapienza, Rome, Italy; 4 University of Sassari, Sassari, Italy

**Keywords:** Scientific productivity, Research output, Database analysis, Research methodology

## Abstract

**Objective:**

This research note aimed to analyze the scientific productivity trends 2015–2019, focusing on the top 30 universities in Europe and United States and on the top 30 private companies—as classified in the SCImago Institutions Ranking. Our hypothesis is that private companies are gaining an increasingly prominent role in the research field, while academia is losing its predominance.

**Results:**

From 2015 to 2019, all universities in Europe and the United States lost positions in the scientific production ranking, while private companies gained positions. These trends seem to be driven mainly by the scientific productivity sub-indicator “Innovation”. These data suggest that the role private companies will play in the future will not be limited to support research economically or influence it from “outside”. Private companies have taken a path that may lead them to directly control all stages of production/communication of knowledge, including research—a role once bestowed on universities. Our data, although preliminary, seem to suggest that, at present, academia risks losing its predominance in the research field. This scenario deserves attention because of the threats it may pose to the independence of research and its role in supporting human equity and sustainable health for all.

## Introduction

Scientific research has always had a fundamental role in promoting health and the progress of society as a whole. A recent editorial published in The Lancet [1] has discussed plausible upcoming research scenarios and outlined a vision of the future in which research could be an instrument for an “equitable, sustainable world with better health for all” [1].

However, we think that also an alternative—more pessimistic—scenario, emerging from an analysis of recent trends in scientific productivity, deserves attention because of the threats it may pose to the independence of research and its role in supporting human equity and sustainable health for all.

In support of this hypothesis, we will (1) provide an analysis of the scientific productivity trends from 2015 to 2019, focusing on the top 30 universities in Europe and the United States and on the top 30 private companies (2) detail the methodology used and its limits, and (3) discuss the implications of our hypothesis and future addresses.

## Main text

### Methods

We have analyzed the scientific productivity trends from 2015 to 2019. First, we used the SCImago institutions ranking [2] to identify (1) the top 30 universities in Europe, (2) the top 30 universities in the United States, and (3) the top 30 private companies operating in Europe and in the US or at the global level. Each year, SCImago classifies academic and research-related institutions based on a composite indicator of scientific productivity. The scientific productivity indicator is calculated based on three sub-indicators: number of published articles and citations (50% of the total score weight), social media visibility (20%), and innovation (30%) [2, 3]. Innovation is the product of scientific publications cited in patents and number of patent applications in the PATSTAT databank [2, 4]. It is a key dimension for evaluating the impact of research both on economy and well-being of individuals.

We calculated the average position (± standard deviation) in the ranking for scientific productivity by group (universities in Europe, universities in the United States, and private companies) for each year from 2015 to 2019. We then calculated the changes of positions in the ranking for scientific productivity by group from 2015 to 2019.

To see if these changes of positions in the ranking for scientific productivity were significantly different between universities in Europe and universities in the United States vs. private companies, we performed a multivariate Kruskal–Wallis test [5–7]. All statistical analysis performed in SAS 9.4 [8].

### Results

In Europe, the top 30 universities were: Oxford, University College London, University of Cambridge, Imperial College London, Swiss Federal Institute of Technology, Catholic University of Leuven, University of Copenhagen, Utrecht University, University of Amsterdam, Kings College London, University of Manchester, University of Edinburgh, Karolinska Institute, Ecole Polytechnique Federale de Lausanne, Sorbonne Universite, Ghent University, Technische Universitat Munchen, University of Groningen, Leiden University, VU University Amsterdam, Università degli Studi di Roma La Sapienza, Aarhus University, Ludwig-Maximilians Universitat Munchen, Erasmus University Rotterdam, Lunds University, Ecole Pratique des Hautes Etudes, University of Helsinki, Universitat Zurich, Uppsala University, and Technical University of Denmark. In the United States, the top 30 universities were: Harvard University, Harvard Medical School, Massachusetts Institute of Technology, Stanford University, Johns Hopkins University, University of Michigan Ann Arbor, University of Washington, University of Pennsylvania, University of California Los Angeles, Columbia University, University of California San Diego, University of California Berkeley, Cornell University, University of California San Francisco, Yale University, Duke University, University of Wisconsin Madison, University of Minnesota Twin Cities, Northwestern University Evanston, University of North Carolina Chapel Hill, University of Maryland Baltimore, New York University, University of Pittsburgh, Ohio State University Columbus, University of California Davis, University of Southern California, University of Chicago, Pennsylvania State University, University of Florida, and Washington University in Saint Louis. The top 30 private companies operating in Europe and in the US or at the global level were: Google, Facebook Inc., Microsoft Corp, Microsoft USA, Samsung Corp, Regeneron Pharmaceuticals Inc, Hoffmann-La Roche Ltd. United States, Hoffmann-La Roche, IBM United States, Genentech Inc., IBM Research, Alphabet Inc, Novartis Institutes for Biomedical Research United States, Microsoft Research Cambridge, MedImmune LLC., Toyota Group, Nokia Corp, Nokia Finland, GlaxoSmithKline United States, Qualcomm Inc USA, Qualcomm Inc, Sanofi United States, Biogen Idec USA, NEC Corp, Biogen Idec, Novartis, GlaxoSmithKline, Panasonic Corp, LG Corporation, and Pfizer Inc. United States.

From 2015 to 2019, all the universities in these groups lost positions in the scientific production ranking. In Europe, universities lost on average − 9.8 ± 74.2 positions, while in the United States − 5.5 ± 21. On the contrary, private companies gained on average + 129.9 ± 143.9 positions.

These trends seem to be driven mainly by one of the scientific productivity sub-indicators, “Innovation”. Indeed, if we analyze this specific sub-indicator, the trends are even more marked. Universities lost − 166.6 ± 60.6 positions on average in Europe, and − 114.7 ± 51.5 positions in the United States; while private companies gained + 116.5 ± 113.7 positions.

On average, the European universities had higher positions than private companies in the ranking in 2015 (99.3 ± 89.6 vs. 238.4 ± 159.7. Instead, in 2019, European universities and private companies had on average similar positions (109.2 ± 45.5 vs. 107.9 ± 51). The scientific productivity trend from 2015 to 2019 was significantly different between these two groups (F-value 11.52, *p* value < 0.0001).

The United States universities had higher positions than private companies in the ranking both in 2015 (42.8 ± 31.8 vs. 238.4 ± 159.7) and in 2019 (46.8 ± 27.5 vs. 107.9 ± 51). However, again, the scientific productivity trend was significantly different from 2015 to 2019 in these two groups (F-value 12.59, p-value < 0.0001).

### Discussion

Our data, although preliminary, seem to suggest that, at present, academia risks losing its central role in the research field, while private companies are gaining prominence.

It is interesting that, among private companies, sectors historically linked to research (e.g., pharmaceutical industries) have lost power in terms of scientific productivity. In contrast, companies specialized in internet-related services, technology, and data analytics have acquired increasing importance in the research field. Alarmingly, some of these companies, at present in the top ranking for scientific productivity, have recently been accused of using the information they gather from individuals indiscriminately and amorally for the purposes of profit-making and, even worst, social control [9, 10].

The trends above analyzed suggest that the role private companies will play in the future will not be limited to support research economically and/or influence it from “outside”. Indeed, companies specialized in internet-related services, technology, and data analytics have taken a path that may easily—or already has—lead them to directly control all stages of production and communication of knowledge, including research—a role once bestowed on universities. This finding is in contradiction with the results of a recent study by Fleming et al. [11], which shows that private companies do not appear to be interested in pursuing (or publishing) basic science and underinvest in research. These results seem supported by a recent study indicating that patents held by private companies rely on high quality research produced by academia [12]. However, these two studies used data up to 2016, while we examined data from 2015 to 2019, thus our results may represent a more recent trend (specifically related to emerging private companies specialized in internet-related services, technology, and data analytics). Another possibility is that these contrasting results are due to the different methodologies used.

Although caution is needed given the preliminary nature of our data, the trends analyzed seem to suggest that, at present, academia is losing predominance in the research field. The vision matured in the nineteenth-century of universities (and science) as expression of free thought, guarantee for rights, and ability to dialogue with power to further human development and social justice is apparently going through a historic crisis.

If the trends analyzed will be confirmed, the pessimistic vision of the Dialectic of Enlightenment [13] may become a reality: Science that was supposed to sustain human emancipation and freedom risks today to be at the service, without any control, of the interests of single companies. In this situation, the space for a free, independent debate among researchers, aimed to foster an equitable, sustainable world, risks to become increasingly limited.

## Limits and future addresses

The present research note discusses the findings of an analysis of trends in scientific productivity. This is a preliminary work using a simple methodology.

The results of our analysis need to be confirmed using a bigger sample size and data from other geographical areas or specific research fields.

Future research should examine all the scientific productivity sub-indicators: not only “Innovation” but also “Number of published articles and citations” and “Social media visibility”.

Furthermore, future studies should investigate how the cooperation between universities and private companies may influence scientific productivity and, in turn, the ranking.

Finally, it would be critical to have data from sources other than SCImago. Currently, SCImago is the only dataset grouping data by research institution, while similar databanks (e.g., Scopus and Web of Science) do not provide this kind of information.

## Data Availability

The datasets used for the analyses are available at the following links; SCImago Institutions Ranking 2019 (https://www.scimagoir.com/rankings.php?year=2013) SCImago Institutions Ranking 2018 (https://www.scimagoir.com/rankings.php?year=2012) SCImago Institutions Ranking 2017 (https://www.scimagoir.com/rankings.php?year=2011) SCImago Institutions Ranking 2016 (https://www.scimagoir.com/rankings.php?year=2010) SCImago Institutions Ranking 2015 (https://www.scimagoir.com/rankings.php?year=2009).
